# Metabolic adaptations to repeated periods of contraction with reduced blood flow in canine skeletal muscle

**DOI:** 10.1186/1472-6793-5-11

**Published:** 2005-07-14

**Authors:** Alan MacInnes, James A Timmons

**Affiliations:** 1 Cardiovascular Research Department, Pfizer Global R&D, St. Louis, MO63017, USA; 2 Department of Physiology and Pharmacology, Karolinska Institute, Stockholm, Sweden

## Abstract

**Background:**

Patients suffering from Intermittent Claudication (IC) experience repeated periods of muscle contraction with low blood flow, throughout the day and this may contribute to the hypothesised skeletal muscle abnormalities. However, no study has evaluated the consequences of intermittent contraction with low blood flow on skeletal muscle tissue. Our aim was to generate this basic physiological data, determining the 'normal' response of healthy skeletal muscle tissue. We specifically proposed that the metabolic responses to contraction would be modified under such circumstances, revealing endogenous strategies engaged to protect the muscle adenine nucleotide pool. Utilizing a canine gracilis model (n = 9), the muscle was stimulated to contract (5 Hz) for three 10 min periods (separated by 10 min rest) under low blood flow conditions (80% reduced), followed by 1 hr recovery and then a fourth period of 10 min stimulation. Muscle biopsies were obtained prior to and following the first and fourth contraction periods. Direct arterio-venous sampling allowed for the calculation of muscle metabolite efflux and oxygen consumption.

**Results:**

During the first period of contraction, [ATP] was reduced by ~30%. During this period there was also a 10 fold increase in muscle lactate concentration and a substantial increase in muscle lactate and ammonia efflux. Subsequently, lactate efflux was similar during the first three periods, while ammonia efflux was reduced by the third period. Following 1 hr recovery, muscle lactate and phosphocreatine concentrations had returned to resting values, while muscle [ATP] remained 20% lower. During the fourth contraction period no ammonia efflux or change in muscle ATP content occured. Despite such contrasting metabolic responses, muscle tension and oxygen consumption were identical during all contraction periods from 3 to 10 min.

**Conclusion:**

repeated periods of muscle contraction, with low blood flow, results in cessation of muscle ammonia production which is suggestive of a dramatic reduction in flux through AMP deaminase.

## Background

Intermittent Claudication (IC) is the symptomatic manifestation of peripheral vascular disease, suffered by millions of patients worldwide. IC is thought to occur when skeletal muscle blood flow fails to increase adequately during walking [1,2] severely limiting the quality of life of the patient [3]. The effectiveness of current treatment strategies for IC is dismal [4-6]. Arguably, this reflects both the current inadequacies of available pharmaceutical medications [4] and the lack of exercise rehabilitation programs [6]. In order to select potential therapeutic targets, we must establish a greater understanding of the physiological responses to repeated periods of skeletal muscle contraction under low blood flow conditions in both health and disease. In both preclinical models and in humans, accelerated muscle fatigue and increased 'anaerobic' ATP regeneration is a feature of limb ischemia [7-11]. This in turn temporally relates to muscle pain and fatigue experienced by patients [2,12-14]. Evidence for underlying muscle damage and degeneration in ambulatory IC patients has been presented [15,16] but such observations may reflect co-morbidities and have not been directly linked to IC symptoms. In the studies by Holm et al [13] muscle tissue from IC patients demonstrated an increased mitochondrial enzyme capacity. In patients with resting ischemia (and loss of mobility) this increased enzymatic activity was no longer observed [13]. To date Type I muscle fiber content has ranged from 35% to 70% and markers of mitochondrial capacity have been elevated, unchanged or reduced; when compared with age matched controls. Overall, the characteristics of muscle tissue obtained from the IC patient gives little clue to the underlying patho-physiology of IC.

When studying muscle metabolism in IC patients, the impact of repeated period of contraction on muscle metabolism has never been considered. Furthermore, no study has examined healthy skeletal muscle during repeated periods of contraction with low blood flow and hence the 'normal' physiological responses of healthy muscle tissue, under such conditions, have not been defined. Short cycles of *complete *ischemia are known to induce a protective response to subsequent prolonged ischemia (preconditioning) [1,17]. There is little evidence that ischemia-reperfusion injury contributes to the symptoms of IC. It is, however, plausible that the metabolic response to contraction adapts during periods of intermittent walking and that this alters the metabolism and function of the muscle. In the present study we characterize for the first time, the metabolic responses of skeletal muscle, exposed to repeated periods of contraction with low blood flow, using the canine gracilis model [18] which reflects the metabolic responses observed in human skeletal muscle under similar circumstances [9]. We were particularly interested to characterize muscle ATP catabolism and ammonia production. In humans, a 1 hour recovery period between two ~3 min maximum knee extension exercise bouts (with normal blood flow) was sufficient to attenuate muscle ammonia production [19]. This observation suggested to us that a 1 hour recovery period may be sufficient to observe an altered regulation of ATP homeostasis. Our investigation revealed that skeletal muscle protects the ATP pool 1 hr following three periods of 'low flow' contraction and this is associated with a complete lack of ammonia efflux from the muscle tissue.

## Results

### General model characteristics

Blood flow was fixed at 15.7 ± 0.6 ml min^-1 ^100 g^-1 ^muscle tissue throughout the study, reflecting a normal resting value but an 80% reduction in the value observed during contraction. Resting muscle tension averaged 444 ± 33 g 100 g^-1 ^muscle and was fixed prior to each contraction period. Resting muscle perfusion pressure ranged from 95 to 111 mmHg (pre-isolation perfusion pressure was ~125 mmHg). During contraction, muscle perfusion pressure ranged from 55 to 59 mmHg. Arterial hemoglobin concentration was 13.8 ± 0.2 g dl and remained stable during the entire period of study (typically 8–10 hours). Control of ventilation, using a respiratory pump and room air, ensured that arterial saturation (SO_2 _%) was maintained at ~100%, while arterial PCO_2 _was stable at 45 ± 1 mmHg for the duration of the study. Arterial ammonia concentration varied between 16 and 24 μM and arterial glucose concentration averaged 6.5 ± 0.1 mM, both remaining stable throughout the study. Arterial lactate concentration was 1.2 ± 0.1 mM and was also stable for the duration of the experiment. Arterial creatine kinase activity (SI unit/L) varied from 40 ± 5 to 59 ± 19 during the course of the study with no clear pattern with respect to time. There was no evidence of any muscle damage at any stage of these experiments. Additional experiments revealed that during 12 hrs of pump mediated perfusion, peak muscle force development, checked utilizing a short burst of 5 Hz stimulation, was consistent over time, further demonstrating the stability of the model (data not shown).

### Muscle contractile function and oxygen consumption

**Figure 1:**
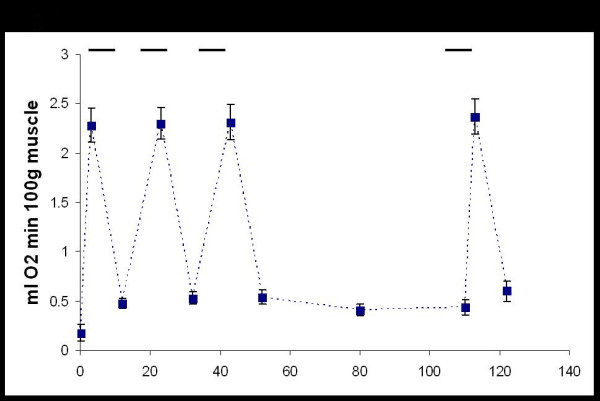
Skeletal muscle oxygen consumption measured at rest and during the 3^rd ^to fourth minute of contraction (this is when a venous blood sample was obtained). Oxygen consumption was calculated from the arterial-venous difference (oxygen content) and muscle blood flow. Oxygen consumption during contraction did not differ across the 4 stimulation periods. The black bars at the top of the graph represent the period of muscle stimulation.

During the first period of contraction, peak tension was 3.38 ± 0.2 kg 100 g^-1 ^muscle tissue. During the second and third periods of contraction, peak tension was reduced to 1.75 ± 0.1 and 1.64 ± 0.1 kg 100 g^-1 ^muscle tissue, respectively. During the fourth period, peak tension recovered to 1.89 ± 0.2 kg 100 g^-1 ^muscle tissue. Time to achieve peak tension was 27.0 ± 1.1 sec during period 1, 73.9 ± 3.1 sec, 87.0 ± 2.0 sec and 75.1 ± 4.9 sec during each successive contraction period. Force rapidly declined during the first minute of contraction during contraction period 1 (1.34 ± 0.08 and 0.84 ± 0.08 kg 100 g^-1 ^muscle tissue at 3 min and 10 min of contraction respectively). During contraction periods 2, 3 and 4, muscle tension production did not differ from contraction period 1 at the 3 min time point (Table 1) or at the end of the 10 min stimulation period (0.84 ± 0.08, 0.81 ± 0.06 and 0.83 ± 0.07 kg 100 g^-1 ^muscle tissue respectively). The lack of difference in muscle tension production at 3 min, across stimulation periods, is consistent with the muscle oxygen consumption data from this time point which indicates a similar metabolic rate (Table 1 and Fig 1).

### Intramuscular metabolites

During the first 10 min period of contraction, muscle ATP concentration fell by ~30% (Table 2). This was accompanied by a 10 fold increase in muscle lactate concentration. Net PCr degradation was substantial, indicating that the majority of muscle fibers were actively contracting at the time of obtaining the 10 min muscle biopsy sample. Following 2 additional periods of muscle contraction, separated by 10 min recovery; with 60 min of recovery following the 3^rd ^period of contraction muscle (See Fig 4), ATP concentration failed to recover to initial levels (~20% lower than the initial concentration). However, during the subsequent contraction period (period 4) ATP levels were maintained during contraction. Furthermore, the extent of the decline in PCr concentration was somewhat reduced (~15%, p = 0.051), while muscle lactate accumulation was ~50% lower than that observed during contraction period 1 (Table 2).

### Muscle ammonia efflux

**Figure 2:**
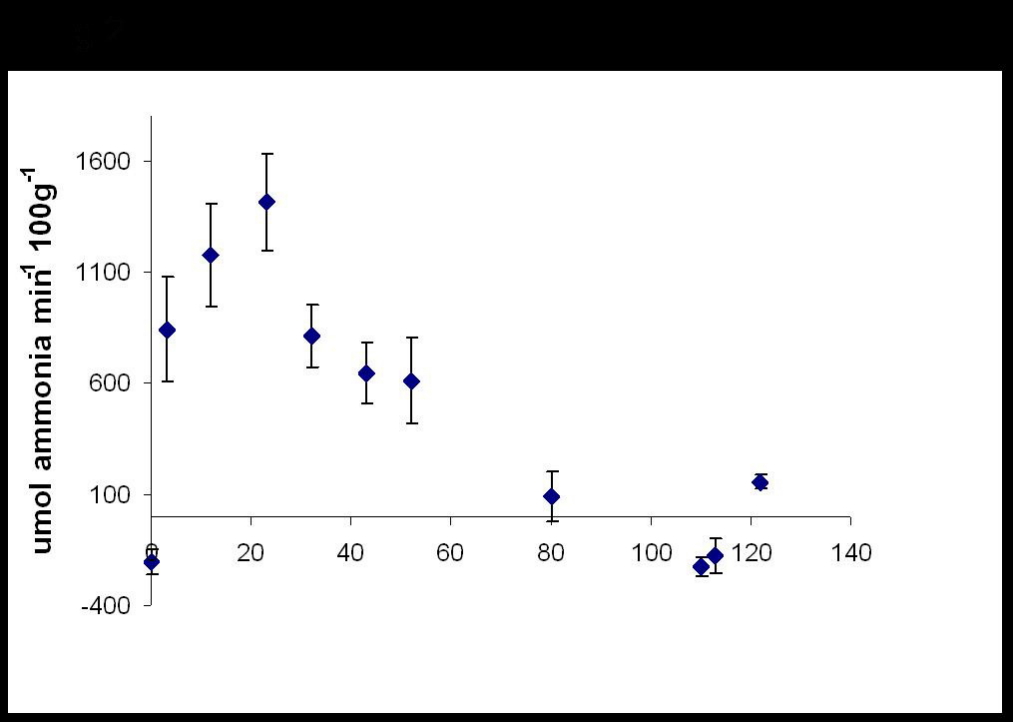
Represents muscle ammonia efflux measured during rest and during the 3^rd ^to fourth minute of contraction (this is when a venous blood sample was obtained) and during the first 2 minutes of recovery. Peak ammonia efflux from the muscle occurred during the 2^nd ^period of muscle contraction. During the fourth period of contraction there was no measurable release of ammonia from the muscle tissue. The black bars at the top of the graph represent the period of muscle stimulation.

Ammonia efflux from the contracting muscle was substantial during contraction periods 1 and 2 (Fig 2) and somewhat reduced during contraction period 3. The substantial ammonia efflux during periods 2 and 3 of contraction indicates that muscle adenine nucleotide catabolism continued during both these contraction periods. It is also interesting to note that muscle ammonia efflux remained elevated during 6 min following the completion of the contraction periods. This is consistent with the idea that temporal efflux reflects ~30% of total ammonia production and therefore there is a delay in the efflux process. During the 1 hr recovery period ammonia efflux returned to the baseline value. During the fourth period of contraction there was a complete lack of ammonia efflux from the muscle. Despite this, peak tension was the second highest out of the 4 periods of stimulation and the 'steady state' tension and oxygen consumption was identical to the other 3 contraction periods (Table 1). It is therefore clear that the reduction in ammonia efflux is not a consequence of lower ATP turnover.

### Muscle lactate efflux

**Figure 3:**
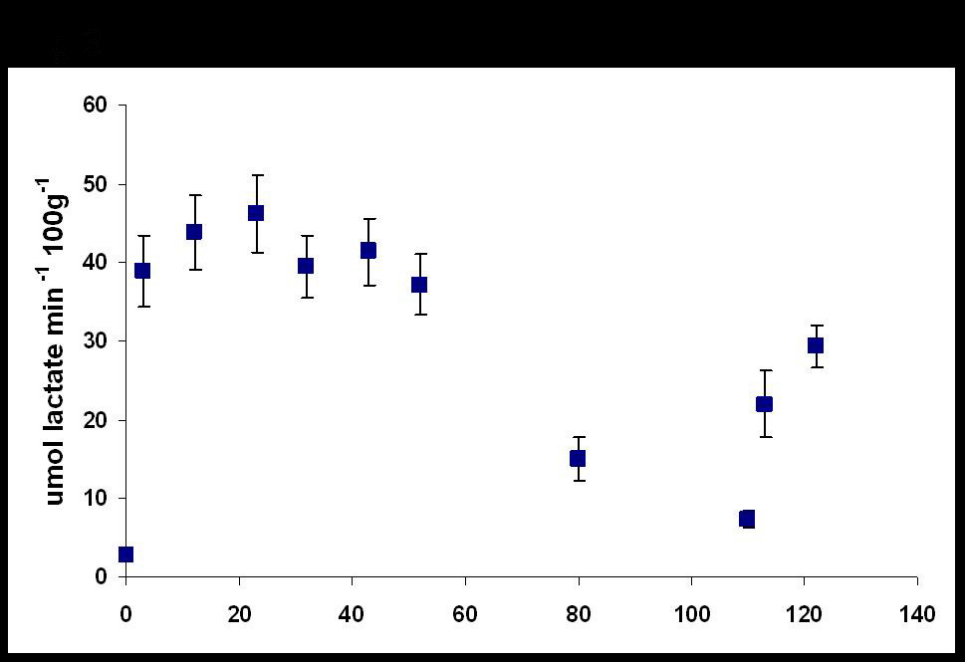
Represents muscle lactate efflux measured during rest and during the 3^rd ^to fourth minute of contraction (this is when a venous blood sample was obtained) and during the first 2 minutes of recovery. Peak lactate efflux muscle was similar during the first 3 periods of muscle contraction. Following 60 min recovery lactate efflux had returned to baseline. The black bars at the top of the graph represent the period of muscle stimulation.

During the first period of muscle contraction there was a 10 fold increase in muscle lactate efflux (Fig 3). Muscle lactate efflux remained elevated during the recovery period and during contraction periods 2 and 3. This indicates a sustained and relatively high glycolytic flux. Muscle lactate efflux during period 4 mirrored the reduction in intramuscular lactate accumulation, being reduced by ~50% (Fig 3). The lactate efflux from the muscle over the first 60 min of contraction/recovery intervals would account for ~33% of total muscle glycogen stores (assuming glycogen as the only source of carbohydrate). As resting canine muscle glycogen concentration is ~250 mmol kg^-1 ^dry muscle [18], (and ignoring glycogen resynthesis during the 60 min recovery period) then muscle glycogen availability would most likely be in excess of 160 mmol kg^-1 ^dry muscle, prior to contraction period 4 and hence can not be considered to be depleted. The significant lactate accumulation and efflux during period 4 supports this interpretation.

## Discussion

There is extensive information on the functional and biochemical characteristics of skeletal muscle in response to a single period of contraction under conditions of low blood flow [10,20,21]. Responses to intermittent 'cycles' of skeletal muscle contraction under conditions of low blood flow have not previously been reported. Our first new observation is that muscle ATP concentration was protected during the fourth period of contraction while the lack of ammonia release indicates that flux through AMP deaminase was dramatically attenuated. This altered biochemical response is particularly apparent if periods 2 and 4 are contrasted. In both cases presented contractile parameters were identical yet ammonia efflux was highest during the 2^nd ^period and completely attenuated during the fourth period of contraction.

During intense muscle contraction reamination of IMP to AMP is thought to be slow [22] and recovery of the adenine nucleotides is thought to be constrained to the post exercise period. During moderate intensity contraction, reduced blood flow to the muscle accelerates ATP degradation [18] to levels observed during supra-maximal exercise [23]. This results in otherwise 'low intensity exercise' having an increased metabolic cost of recovery. The determinants of recovery following ischemia reflect the oxidative capacity of the muscle tissue, including oxygen delivery, but may also include muscle ribose-5-phosphate availability [24]. In the present study we directly assessed ATP degradation during the first and fourth periods of contraction and assessed flux through AMP deaminase indirectly, during each bout, by measuring ammonia efflux. Graham et al [25] demonstrated that muscle ammonia efflux reached a maximum of ~300 μmol per min during intense one legged cycling, reflecting the net adenine nucleotide catabolism in the muscle. In the present study a much higher peak ammonia efflux was observed (1500 μmol min^-1 ^100 g^-1 ^versus 314 μmol per min per leg) indicating that a relative lack of blood flow may be a more potent stimulus to adenine nucleotide loss than the intensity of muscle contraction (absolute ATP turn-over).

### Mechanisms explaining a reduced ammonia production

Given the short recovery period between the first two periods of contraction, pre-contraction [ATP] is likely to have been lowered prior to contraction period 2, yet ammonia efflux was extremely high. This indicates that the 20% reduction in resting [ATP], prior to the fourth period of contraction, cannot explain the complete lack of ammonia production. PCr utilization was modestly reduced during the fourth period of contraction, when compared with period 1 (p = 0.051). Although not substantial, it is suggestive of a more rapid increase in ATP derived from oxidative phosphorylation [8-11,26]. A more rapid increase in the rate of oxidative ATP regeneration ensures that ATP homeostasis is better maintained [10]. Given that there was a 1 hour recovery period, prior to the fourth period of contraction, our data also provide an indication that muscle metabolism is altered for a substantially longer period of time following prior exercise than presently assumed.

Alterations in substrate utilization can influence muscle pH during contraction, and hence the activation of AMP deaminase, See [27]. There was a substantial reduction in muscle lactate accumulation and a measurable reduction in muscle lactate efflux. The reduced lactate production indicates that glycolytic flux was attenuated and hence (Table 2 and Fig 3) it is reasonable to suggest that muscle pH did not decline as much during the fourth period of contraction when compared with any of the first 3 periods of contraction. Given that flux through AMP deaminase – as assessed by muscle ammonia efflux and the change in muscle [ATP] – was essentially zero during the fourth period of contraction it is unlikely that the attenuation of muscle acidosis fully explains the altered regulation of ATP catabolism but clearly it could have contributed. Less accumulation of substrate for AMP deaminase or specific inactivation of AMP deaminase both could explain stabilization of the adenine nucleotide pool and reduced ammonia production [27].

Although there are no previous studies addressing the metabolic response of skeletal muscle to repeated contractions with low blood flow, there are a limited number of intermittent exercise studies (with high blood flow) which can be compared with the present data [19,28]. During four bouts of ~3 mins of isotonic contraction with ~3 min of rest between each successive bout, there was a successive reduction in muscle pH and PCr utilization [28]. In this case, it is plausible that the reduced PCr utilization reflected a greater resting muscle acetyl group availability [29]. Graham *et al *[19,25] provide insight into the impact of prior exercise on ammonia production. They demonstrated that a period of exhaustive exercise carried out 24 hr prior to 3 min intense contraction attenuated muscle ammonia production by ~50% while the total work done was similar [19]. This effect was independent of muscle glycogen status, which remained above 200 mmol kg^-1 ^dry muscle tissue, under all conditions. It is therefore conceivable that the metabolic response to walking, in IC patients, could be *greatly *altered by earlier walking activity, impacting on the outcome of any clinical assessment and almost certainly complicating the 'disease' phenotype when studied in a laboratory setting.

ATP loss can exceed 40% even during a single period of contraction with low blood flow [20] and thus the cessation of ammonia efflux during period 4 is highly unlikely to be a consequence of the 20% lower baseline [ATP]. Under the present experimental conditions amino acid deamination would represent only a small percentage of the observed muscle ammonia efflux [30,31]. It could also be suggested that glutamine synthetase (GS) may have consumed ammonia, for example, during the fourth period of contraction. Again, given the rate of ammonia production observed, we feel that it is highly unlikely that GS activity is relevant for our observations. Finally, reduced ammonia production by AMP deaminase may have also reflected the activation of alternative routes of AMP metabolism, including 5'nucleotidase. The maximal capacity of 5'nucleotidase is much lower than AMP deaminase and since [ATP] did not significantly decline during the fourth period of contraction we also feel that this is not a likely explanation for the attenuation of ammonia production and stabilization of muscle [ATP].

## Conclusion

We can hypothesize that following 3 periods of contractions with low blood flow, adenine nucleotides loss is prevented by an endogenous mechanism, involving modification of AMP deaminase activity. The present study also provides support for the idea that flux through AMP deaminase is not essential for continued muscle contraction while inhibition of AMP deaminase may represent a potential strategy for protecting muscle metabolism in peripheral vascular disease patients [32-35].

## Methods

### Surgical procedures

After an overnight fast, each female dog (Animal Breeding Unit, PGRD UK, n = 9) was anaesthetized with sodium pentobarbitone (45 ± 1 mg/kg body mass (bm) followed by continuous infusion at 0.10 ± 0.01 mg/kg/min i.v. (Sagatal, Rhône Merieux, Harlow, UK). The trachea was intubated and the dogs were artificially ventilated (24 cycles/min, tidal volume = 13 – 15 ml/kg). The right brachial artery was cannulated and systemic blood pressure was recorded using a pressure transducer and chart recorder. The left brachial artery and vein were cannulated for collection of arterial blood samples for monitoring of blood parameters (e.g. pH, hemoglobin (Hb), PCO_2 _and PO_2_) using the Radiometer ABL 625GL (Copenhagen) and venous infusion of heparin, bicarbonate or saline. A gracilis muscle was vascularly isolated, leaving only the main arterial and venous blood flows intact. The distal tendon of the muscle was attached to an isometric force transducer (Grass FTC 10, Quincy, Medfield, MA, USA). The popliteal artery was catheterized for recording gracilis muscle perfusion pressure. Heparin was infused following surgery, (Multiparin, 1 U/kg/bm/min) for the duration of the experiment. The femoral artery supplying each gracilis muscle was cannulated proximally and distally and attached sequentially to a perfusion pump (Minipuls 3, Gilson, Villiers le bel, France). The muscle blood flow was fixed by setting the perfusion pump at 6.1 ± 0.1 ml/min. We have previously established this as being similar to the resting flow rate in the gracilis muscle [18]. This flow rate was maintained for the duration of the experiment and equates to ~20% of the normal flow observed when this muscle was stimulated to contract with the blood supply intact at 3 Hz twitch. All experiments were carried out in full accordance with the United Kingdom Home Office Animals (Scientific Procedures) Act of 1986, and approved by the local ethics committee.

### Muscle stimulation parameters and blood sampling

**Figure 4:**
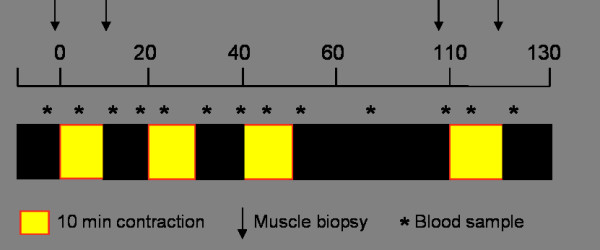
Overview of experimental design.

The resting length of the muscle was altered to obtain a standard tension of ~400 g/100 g muscle. After a 20 min equilibrium period, baseline values were collected (blood samples and muscle biopsies) and then muscle contraction was induced via electrical stimulation of the obturator nerve (Grass S88 stimulator, Quincy, Medfield, MA, USA). Square-wave impulses of 0.1 msec duration, 5 Hz frequency and a supramaximal voltage (10 V), resulting in complete muscle fiber recruitment and peak twitch tension, were applied for 3 periods of 10 min (with 10 min recovery) followed by 1 hour recovery and then a fourth 10 min period of contraction (as shown in Figure 4). Arterial blood samples were taken throughout the experimental period (for monitoring the stability of the animal) and did not demonstrate any significant variation over time. Venous blood samples were obtained to calculate oxygen uptake and ammonia and lactate efflux. The difference between arterial concentration and venous concentration was used, along with the blood flow parameter, to make such calculations. Venous samples were obtained at rest, just prior to the onset of contraction, and at 3 min after the onset of contraction and 6 min after the cessation of contraction. In each case venous sampling took appropriately 30 sec.

### Metabolite analysis

Blood gases, whole blood lactate and glucose were measured using the Radiometer ABL 625GL (Copenhagen). The analyzer was calibrated on a daily basis according to the manufactures instructions. Plasma ammonia was determined in 1-ml blood samples treated according to the standard instructions (Ammonia-Test Wako, Wako Chemicals, Osaka, Japan), and indophenol was measured using a spectrophotometer at a wavelength of 630 nm (UV-2400PC, Shimadzu, Tokyo, Japan). All biopsy samples were stored in liquid nitrogen. Subsequently, a portion was freeze dried, dissected free from visible connective tissue and blood, powdered and extracted in 0.5 M perchloric acid containing 1 mM EDTA. Following centrifugation, the supernatant was neutralized with 2.2 M KHCO_3 _and used for determination of ATP, phosphocreatine (PCr), creatine and lactate as previously described [18] with the exception that a 96-well plate format was utilized using a Spectromax Plus (Molecular Devices). All data are reported as mean ± SE. Analysis of variance was utilized to determine significant changes with respect to time, followed by post-hoc significance testing. A paired t-test was used to compare the intramuscular metabolic differences. Significance was accepted at the 5% level.

## Abbreviations

ATP adenosine triphosphate

AMP adenosine monophosphate

IMP inosine monophosphate

PCr phosphocreatine

IC Intermittent Claudication

GS glutamine synthetase

## Authors' contributions

JAT originated the study idea and JAT and AM designed and carried out the study. The article was written by AM and JAT.

## Figures and Tables

**Table 1:** 

	**Period 1**	**Period 2**	**Period 3**	**Period 4**
	
**VO_2_**	2.3 ± 0.17	2.3 ± 0.16	2.3 ± 0.18	2.4 ± 0.18
**Art [O_2_]**	17.9 ± 0.28	17.9 ± 0.3	17.9 ± 0.3	17.6 ± 0.4
**Art [CO_2_]**	48.5 ± 0.6	48.5 ± 0.4	48.4 ± 0.5	47.8 ± 0.7
**Ven [O_2_]**	3.69 ± 1.02	3.29 ± 0.86	3.19 ± 0.92	2.65 ± 0.73
**Ven [CO_2_]**	64.9 ± 1.4	59.0 ± 0.76	58.6 ± 1.16	58.9 ± 1.37
**Ven Sat %**	18.8 ± 5.3	16.8 ± 4.5	16.3 ± 4.8	14.2 ± 4.1
**Tension**	1.34 ± 0.08	1.28 ± 0.09	1.20 ± 0.11	1.33 ± 0.12
**Ammonia efflux**	843 ± 237	1413 ± 217	646 ± 135	-175 ± 76
**Lactate efflux**	38.9 ± 4.6	46.2 ± 4.9	41.4 ± 4.3	22.0 ± 4.3

Represents metabolic and function data during the 3^rd ^and fourth minute of contraction. Muscle oxygen consumption (VO_2_) units are ml O_2 _min^-1 ^100 g^-1 ^wet tissue and value represents the arterial-venous oxygen content difference multiplied by blood flow. Oxygen (O_2_) and Carbon dioxide (CO_2_) units are ml/DL where 'art' = arterial and 'Ven' equal venous. Sat = Saturation. Tension produced is kg 100 g^-1 ^wet tissue at 3 min into the stimulation protocol. Muscle lactate efflux value represents the venous-arterial [lactate] difference multiplied by blood flow. Venous blood sampling was initiated at the 3^rd ^minute and took appropriately 30 sec to obtain.

**Table 2:** 

	**Period 1**		**Period 4**	
	**Rest**	**Contraction**	**Rest**	**Contraction**
	
ATP	24.7 ± 1.5	17.4 ± 1.6*	19.7 ± 1.1†	18.7 ± 1.2
PCr	86.1 ± 3.4	13.9 ± 4.0*	95.7 ± 6.0	35.1 ± 7.3*
Cr	55.3 ± 3.4	127.5 ± 3.4*	44.7 ± 4.5	105.3 ± 4.7*
Lactate	9.7 ± 1.6	105.3 ± 9.0*	9.4 ± 2.6	47.9 ± 8.9*†

Intramuscular metabolites prior to and at the end of the first and fourth period of muscle contraction. All metabolites are mmol kg^-1 ^dry muscle. *indicates significant change from rest, P < 0.05. †indicates significant difference between period 1 and period 4, P < 0.05. For phosphocreatine, the difference between period 1 and period 4 resulted in a p value of 0.051.
